# Cardiopulmonary collapse in frail patients treated with cemented and uncemented hemiarthroplasty

**DOI:** 10.1007/s00068-025-02856-0

**Published:** 2025-04-23

**Authors:** Thomas P. Bosch, Max P. L. van der Sijp, Pieta Krijnen, Arthur H. P. Niggebrugge, Rachid Mahdad, Inger B. Schipper

**Affiliations:** 1 Department of Trauma Surgery, Leiden University Medical Centre, P.O. Box 9600, 2300 RC Leiden, the Netherlands; 2 Department of Surgery, Haaglanden Medical Centre, P.O. Box 432, 2501 CK The Hague, the Netherlands; 3 Network Acute Care West, P.O. Box 9600, 2300 RC Leiden, the Netherlands; 4 Department of Orthopedic Surgery, Alrijne Hospital, P.O. Box 4220, 2350 CC Leiderdorp, the Netherlands

**Keywords:** Bone cement implantation syndrome, Cardiopulmonary collapse, Cementation, Femoral neck fractures, Hemiarthroplasty, Intraoperative complications, Perioperative management

## Abstract

**Background:**

Peri-operative cardiopulmonary collapse (CPC) poses an increased risk of in-hospital mortality, especially in frail patients. Bone Cement Implantation Syndrome (BCIS) is CPC following, cemented, arthroplasty, characterized by hypoxia and/or hypotension. The main objective of this study was to evaluate the association between cemented hemiarthroplasty and CPC, in patients with a femoral neck fracture (FNF) and increased pre-operative risk, and identify other risk factors for cardiopulmonary collapse.

**Methods:**

This retrospective cohort study included patients with a FNF treated with a cemented or uncemented hemiarthroplasty, aged ≥ 80 years, with ASA score ≥ 3 and ≥ 1 cardiac or pulmonal comorbidity. CPC was defined as hypoxia/hypotension grade ≥ 2 according to Donaldson’s criteria. Multivariable logistic regression analysis was used to adjust for confounding in the relation between cemented hemiarthroplasty and CPC, and to identify other risk factors for CPC in patients with a cemented hemiarthroplasty.

**Results:**

The incidence of CPC was 51.1% in 221 cemented hemiarthroplasty patients compared to 23.3% in 73 uncemented hemiarthroplasty patients (p < 0.001). The use of cement increased the risk for CPC almost threefold (adjusted odds ratio [aOR] 2.87, 95% confidence interval [CI] 1.46–5.64). Preoperative reduced left ventricle ejection fraction (aOR 3.03, 95% CI 1.50–6.14) was another independent risk factor for CPC.

**Conclusion:**

Cementation increases the risk of CPC in frail FNF patients treated with hemiarthroplasty. Emphasis on euvolemia and avoidance of excessive pressurization, and careful consideration of an indicated cemented hemiarthroplasty in frail hip fracture patients may be advised for patients with an increased preoperative risk for BCIS.

## Introduction

The global incidence of hip fractures is estimated to reach 6.3 million in 2050 [1]. Approximately 50% of these hip fractures will be treated with hemiarthroplasty. In-hospital mortality following hemiarthroplasty is approximately 2% [2]. A significant part of in-hospital mortality can be attributed to perioperative cardiopulmonary collapse, defined as perioperative acute hypotension and/or hypoxia [3–7]. Patients treated with cemented hemiarthroplasty are 1.5 times as likely to die perioperatively compared to patients treated with uncemented hemiarthroplasty [2]. Cardiopulmonary collapse following cementation of the hemiarthroplasty is referred to as severe bone cement implantation syndrome (BCIS). More specifically, BCIS is defined as the occurrence of acute hypoxia and/or hypotension around the time of cementation, prosthesis insertion or reduction of the joint in a patient undergoing bone surgery. Severe BCIS, also known as BCIS grade ≥ 2 (Table 1) occurs in about 7% of all patients treated with cemented hemiarthroplasty and poses an increased risk of mortality, especially in frail patients [4, 5, 8, 9].

**Table 1:** Classification of Bone Cement Implantation Syndrome according to Donaldson et al. [8]

Grade 0	No perioperative desaturation (stable oxygen saturation ≥ 94%) or hypotension (no decrease in systolic arterial pressure > 20%)
Grade 1	Mild hypoxia (arterial oxygen saturation < 94%) or mild hypotension (a decrease in systolic arterial pressure > 20%)
Grade 2	Severe hypoxia (arterial oxygen saturation < 88%) or severe hypotension (a decrease in systolic arterial pressure > 40%)
Grade 3	Cardiovascular collapse requiring cardiopulmonary resuscitation and/or resulting in death within 48 h after surgery

The generally accepted theory on the etiology of BCIS is that, mostly fat, embolisms are forced into the systemic blood circulation by intramedullary pressurization, caused by the expanding intramedullary cement. Fat embolisms have been observed using transesophageal echocardiography in more than 50% of patients undergoing cemented total hip arthroplasty [10]. These embolisms follow the physiological venous return and may cause lung embolization when entering the pulmonary circulation [8]. Other factors that may increase the risk of developing cardiopulmonary collapse include old age, pre-existing poor physical reserves, cardiopulmonary comorbidities, pulmonary hypertension, and osteoporosis [4, 8, 11]. A recent retrospective study found the creation of a femoral borehole to be a protective factor for BCIS, which seems to be in line with the theorized pathophysiology [9].

Risk factors for cardiopulmonary collapse are more prevalent in patients treated with hip arthroplasty compared to patients treated with other types of arthroplasties, as hip fracture patients are generally old and often have multiple comorbidities [12, 13]. The higher incidence of cement-induced cardiopulmonary collapse (BCIS) in hip fracture patients may be attributed to the fact that the femur is the largest human bone with a relatively large intramedullary surface area that is pressurized during cementation. Moreover, 70% of the hip fracture patient population has osteoporosis. The enlarged porous cavities of osteoporotic bone form more common surface area with blood circulation which further elevates the risk of embolism formation in this specific patient population [13–16].

The current consensus, based on numerous large studies, is that patients with a femoral neck fracture (FNF) treated with cemented hemiarthroplasty generally experience better clinical outcomes than to those treated without cement [3, 4, 6, 17]. However, this may not be the case for frail patients, since the use of cement may substantially increase the risk of cardiopulmonary collapse and subsequent morbidity and mortality. The aim of this observational study was to assess the association of cardiopulmonary collapse in FNF patients with a high a priori risk for this condition, treated with cemented hemiarthroplasty versus those with uncemented hemiarthroplasty. Furthermore, potential other risk factors for cardiopulmonary collapse after cemented and uncemented hemiarthroplasty in this patient population were evaluated, as well as the association of cemented hemiarthroplasty with surgical complications and in-hospital mortality. The findings of this study may help clinicians to assess patients’ perioperative risks and manage these accordingly.

## Methods

This retrospective cohort study was performed and reported according to the ‘Strengthening the Reporting of Observational Studies in Epidemiology (STROBE)’ statement guidelines [18]. Personal data were handled according to the Dutch Personal Data Protection Act. The institutional Medical Research Ethics Committee approved the study (METC Leiden-The Hague-Delft; protocol number G20.199) and waived the requirement for written informed consent.

### Patients

Data of patients with a FNF aged ≥ 80 years, and with an American Society of Anaesthesiologists (ASA) classification score of ≥ 3 and at least one cardiac or pulmonary comorbidity were collected from the medical records of two large hospitals in the Netherlands. One cohort (cemented) consisted of patients with FNFs who were routinely treated between December 2016 and March 2022 with cemented hemiarthroplasty in the Haaglanden Medical Centre in the Hague. The second cohort (uncemented) was treated between December 2015 and August 2017 with uncemented hemiarthroplasty, the standard of care at that time in the Alrijne Hospital in Leiderdorp.

The Taperloc complete in a standard and high offset version with a monopolar head, both produced by Zimmer Biomet, was used for the uncemented hemiarthroplasty.

For cemented hemiarthroplasty, the self-centering, 3-point-contact anchorage cemented CCA straight stem prosthesis was used, in a standard or lateral version combined with a short or medium hemiprosthesis head produced by Mathys Medical®. Palacos Gentamicine from Heraeus Medical was used as bone cement. The cement was prepared using the vacuum mixing technique. Pulsed lavage was used before cementation. All surgeons used a cement plug, and none made a borehole in the distal femur. In all patients, pressurization was achieved, most commonly by using a retrograde cement application with a cement gun.

### Outcomes and other study parameters

The primary outcome of this study was the incidence of cardiopulmonary collapse within 48 h after operation, defined as the clinical conditions reflecting BCIS grade 2 or 3 according to the classification proposed by Donaldson et al. (Table 1) [8]. The perioperative measurements of arterial oxygen saturation and arterial blood pressure were reviewed. The first measured blood pressure in the operation room, well before the start of surgery, was used together with the lowest systolic blood pressure and the peripheral capillary saturation levels after prosthesis insertion to determine the grade of BCIS.

Secondary outcomes were surgical complications and in-hospital mortality. Surgical complications included incorrect positioning of the prosthesis, prosthesis loosening or dislocation, operative periprosthetic fracture, wound infection, prothesis luxation and any surgical revisions.

Other study parameters concerned patient characteristics and treatment related aspects. These variables were selected because they have been described previously as potential confounders for cement induced cardiopulmonary collapse in literature [4, 5, 8].

### Data analyses

All data analyses were performed using IBM SPSS statistics software for Windows version 25.0. P-values < 0.05 were considered statistically significant. Demographic and clinical characteristics of the patient groups were described using summary statistics and compared by univariable analysis. Continuous variables were compared between patient groups using an unpaired t-test for normally distributed data or using the Mann–Whitney U Test for skewed data (Kolmogorov–Smirnov test, p < 0.05). Categorical variables were compared using the Chi-square test if the groups were sufficiently large (expected cell counts > 5) or Fisher’s exact test if this condition was not met. Risk factors for cardiopulmonary collapse were identified in a multivariable logistic regression analysis that included all potential risk factors with p < 0.20 in the univariable analysis. In two other multivariable logistic regression analyses, the association of cemented fixation with the occurrence of surgical complications and in-hospital mortality was assessed. All patient and treatment characteristics that differed between the two cohorts in the univariable analysis with p < 0.05, were entered in these multivariable models to adjust for potential confounding. Multiple imputation of missing data was used in all multivariable analyses to reduce the risk of bias.

### Sample size

No formal sample size calculation was performed for this observational study, because in literature data on the incidence of cardiopulmonary complications in this specific study population of patients with a high a priori risk of cardiopulmonary collapse, are lacking.

## Results

Nine of the 303 eligible patients were excluded due to incomplete perioperative measurements. Of the 294 included patients, 73 were treated with uncemented hemiarthroplasty and 221 patients with cemented arthroplasty. The mean follow-up time was 41.5 months. The median age of all patients was 88 years, and the majority were women (63.9%) (Table 2).

**Table 2:** Patient characteristics and treatment aspects per fixation method

Variable	Total (n = 294)	Uncemented (n = 73)	Cemented (n = 221)	P-value
Patient characteristics				
Age, y, median (IQR)	88 (84–91)	86 (80–92)	88 (80–96)	0.26
Sex, f, n (%)	188 (63.9)	46 (63.0)	142 (64.3)	0.85
Body mass index, kg/cm^2^, median (IQR)	23.5 (21.1–26.2)	24.5 (18.7–30.4)	23.5 (18.3–28.6)	0.02
ASA classification, n (%)				
3	253 (86.1)	57 (78.1)	196 (88.7)	0.02
4	41 (13.9)	16 (21.9)	25 (11.3)	
Cardiac comorbidity^a^, n (%)	266 (90.5)	69 (94.5)	197 (89.1)	0.18
Pulmonary comorbidity^b^, n (%)	102 (34.9)	28 (38.4)	74 (33.8)	0.48
Dementia^c^, n (%)	141 (48.0)	24 (32.9)	117 (52.9)	0.003
CVA/TIA, n (%)	85 (28.9)	24 (32.9)	61 (27.6)	0.39
Chronic renal failure, n (%)	42 (14.3)	11 (15.1)	31 (14.0)	0.83
Dislocated fracture, n (%)	241 (82)	70 (95.9)	171 (77.4)	< 0.001
Treatment aspects				
Hours till operation, median (IQR)	26 (19–53)	42 (24–50)	23 (1–53)	< 0.001
General anesthesia^d^, n (%)	142 (49)	30 (41.7)	112 (51.4)	0.15
Use of inotropy^e^, n (%)	260 (90.6)	51 (73.9)	209 (95.9)	< 0.001

*IQR* interquartile range

^a^Defined as ischemic heart disease (including: history of MI; history of positive exercise test; current chest pain considered due to myocardial ischemia; use of nitrate therapy; ECG with pathological Q waves; angina pectoris), congestive heart failure (including: pulmonary edema; bilateral rales or S3 gallop; paroxysmal nocturnal dyspnea; chest x-ray (CXR) showing pulmonary vascular redistribution), abnormal left ventricular ejection fraction (LVEF, including: heart valve stenosis, history of heart valve replacement), valve insufficiency (including heart valve replacement), arrhythmia (including an implanted pacemaker/ICD) and/or the use of cardiac medication (B-blockers, diuretics, ACE inhibitors)

bDefined as chronic obstructive pulmonary disease (COPD, including abnormal target hemoglobin saturation), other causes of reserved lung function and/or the use of pulmonary medication (bronchodilators, steroids for pulmonary disease)

cDefined as a previously diagnosed form of dementia or a Six Item Cognitive Impairment Test (6-CIT) score of ≥ 11 upon admission)

^d^General vs spinal anesthesia

^e^Defined as the use of phenylephrine, norepinephrine, epinephrine, vasopressin, milrinone, dobutamine and dopamine

The patients in the uncemented group had a higher median Body Mass Index (24.5 vs 23.5, P = 0.02), were classified more often as ASA 4 (21.9% vs 11.3%, P = 0.02), had less frequently dementia (32.9% vs 52.9%, P = 0.003), had more often a dislocated fracture (95.9% vs 77.4%, P < 0.001) and were less frequently treated with inotropy during surgery (73.9% vs 95.9%, P < 0.001). The median time till operation was longer in the uncemented group (42 vs 23 h, P < 0.001; Table 2).

### Incidence of cardiopulmonary collapse and risk factors

The overall incidence of cardiopulmonary collapse was 44.2% (130/294 patients), 51.1% in 221 cemented hemiarthroplasty patients compared to 23.3% in 73 uncemented hemiarthroplasty patients (p < 0.001). Besides cemented hemiarthroplasty (adjusted Odds Ratio [aOR] 2.87; 95% confidence interval [CI] 1.46–5.64), another independent risk factor for cardiopulmonary collapse was having an abnormal left ventricle ejection fraction (LVEF) (aOR 3.03; 95% CI 1.50–6.14) (Table 3). Having had a CVA/TIA decreased the risk of cardiopulmonary collapse (aOR 0.50; 95% CI 0.29–0.88).

**Table 3:** Characteristics and treatment aspects associated with cardiopulmonary collapse in patients treated with cemented and uncemented hemiarthroplasty

	Cardiopulmonary collapse		Unadjusted OR (95% CI)	P value	Adjusted* OR (95% CI)	P value
	No (n = 164)	Yes (n = 130)				
Patient characteristics						
Use of cement, n (%)	108 (65.9)	113 (86.9)	3.45 (1.89–6.30)	** < 0.001**	2.87 (1.46–5.64)	**0.002**
Age, y, median (IQR)	87.0 (84.0–91.0)	88.0 (84.8–92.0)	1.03 (0.98–1.08)	0.27		
Sex, f, n (%)	103 (62.8)	85 (65.4)	1.12 (0.69–1.81)	0.65		
BMI, kg/cm^2^, median (IQR)	23.73 (20.94–26.32)	23.41 (21.19–26.05)	0.97 (0.92–1.03)	0.35		
ASA Classification (4), n (%)	24 (14.6)	17 (13.1)	0.88 (0.45–1.71)	0.70		
Cardiac comorbidity, n (%)	150 (91.5)	116 (89.2)	0.77 (0.36–1.69)	0.52		
Cardiac ischemia	57 (34.8)	42 (32.3%)	0.90 (0.55–1.46)	0.66		
Decompensation cordis	15 (9.1%)	15 (11.5%)	1.30 (0.61–2.76)	0.50		
Abnormal LVEF	16 (9.8)	30 (23.1)	2.78 (1.44–5.34)	**0.002**	3.03 (1.50–6.14)	**0. 002**
Heart valve insufficiency	24 (14.6)	18 (13.8)	0.94 (0.49–1.81)	0.85		
Cardiac arrythmia	91 (55.5)	75 (57.1)	1.09 (0.69–1.74)	0.71		
Pulmonal comorbidity, n (%)	53 (32.3%)	49 (38.3%)	1.23 (0.80–2.11)	0.29		
COPD	37 (22.6%)	36 (27.7%)	1.32 (0.77–2.24)	0.31		
Other reserved lung function	17 (10.4)	15 (11.5%)	1.13 (0.54–2.35)	0.75		
Dementia, n (%)	78 (47.6)	63 (48.5)	1.04 (0.65–1.64)	0.88		
CVA/TIA, n (%)	58 (35.4)	27 (20.8)	0.48 (0.28–0.82)	**0.007**	0.50 (0.29–0.88)	**0.02**
Chronic renal failure, n (%)	25 (15.2%)	17 (13.1%)	0.84 (0.43–1.63)	0.47		
Treatment aspects						
Hours till operation, median (IQR)	30 (20–46)	24 (18–39)	0.98 (0.97–0.99)	**0.004**	0.99 (0.97–1.01)	0.07
Use of inotropy, n (%)	140 (87.5)	120 (94.5)	2.45 (1.00–5.99)	**0.05**	1.36 (0.50–3.71)	0.55

All p values which were deemed significant (p < 0.05) were highlighted using bold

*LVEF* left ventricle ejection fraction, *COPD* Chronic Obstructive Pulmonary Disease, *OR* odds ratio, *CI* confidence interval

*Adjusted for significant (p < 0.20) variables in the univariate analysis

### Other clinical outcomes

In-hospital mortality in the uncemented cohort tended to be lower than in the cemented cohort (1.4% vs 7.7%, P = 0.051; OR 6.0, 95% CI 0.8–45.9; Table 4). Also after correction for potential confounding, there was no statistically significant effect of cementation on in-hospital mortality (aOR 5.6, 95% CI 0.7–48.9). The incidence of surgical complications was comparable in the uncemented and cemented cohort (2.7% vs 3.2%, P = 0.85; OR 1.2, 95% CI 0.2–5.7). The effect of cementation on surgical complications remained absent after correction for potential confounding (aOR 0.6, 95% CI 0.1–3.7; Table 4).

**Table 4:** Association of fixation technique (uncemented vs cemented) with in-hospital mortality

Type of complication	Uncemented, n (%)	Cemented, n (%)	Unadjusted OR (95% CI)	P	Adjusted* OR (95% CI)	P
In-hospital mortality	1 (1.4)	17 (7.7)	6.00 (0.78–45.90)	0.051	5.57 (0.66–48.94)	0.11
Surgical complications	2 (2.7)	7 (3.2)	1.16 (0.24–5.72)	0.85	0.62 (0.10–3.73)	0.60

*OR* odds ratio; *CI* confidence interval;

*Adjusted for statistically significant (p < 0.05) differences in baseline characteristics (see Table 2)

## Discussion

This study is to our knowledge the first to investigate the association of cemented hemiarthroplasty with cardiopulmonary collapse, combined with the identification of potential risk factors for cardiopulmonary collapse, in FNF patients with a high preoperative risk. The findings of the study suggest that cemented hemiarthroplasty increases the risk of perioperative cardiopulmonary collapse for these frail patients about threefold. Abnormal left ventricle ejection fraction was identified as another independent risk factor of cardiopulmonary collapse, while CVA or TIA in the medical history of patients decreased this risk. Cementation did not show an association with the incidence of surgical complications and in-hospital mortality.

Previous studies have compared the clinical outcomes of cemented or uncemented arthroplasty in the general hip fracture population. The general consensus is that both treatments are adequate, but cemented hemiarthroplasty is becoming the dominant treatment as patients treated with cemented arthroplasty experience better functional outcomes, less residual pain and fewer implant-related complications and revisions [3, 6, 17, 19]. However, several studies did find a difference in perioperative mortality favoring uncemented hemiarthroplasty [3, 6, 17, 19]. Our study indicates that frail femoral neck fracture patients with an a-priori increased risk of cardiopulmonary collapse are about three times more likely to develop cardiopulmonary collapse if treated with cemented hemiarthroplasty.

In literature, some studies report on mortality in relation to cardiopulmonary collapse due to cementation in the general hip fracture population. One study concluded that severe BCIS (grade > 2), equivalent to cardiopulmonary collapse in the present study, leads to a 16-fold increase in 30-day postoperative mortality in patients treated with cemented hemiarthroplasty [5]. Another study confirmed the association of severe BCIS with 30-day mortality in multiple types of cemented arthroplasties, including hemiarthroplasty [11]. A recently conducted meta-analysis concluded that patients treated with cemented hemiarthroplasty had a 60% higher 2-day mortality rate compared to patients treated with uncemented hemiarthroplasty [2]. The studies reporting on the difference in perioperative mortality suggested that this increased mortality risk may be caused by cement induced cardiopulmonary collapse in patients treated with cemented hemiarthroplasty. [2, 6, 20–23].

For frail femoral neck fracture patients with an a-priori increased risk of cardiopulmonary collapse, our study indicates that fixation without cement might be beneficial in terms of decreased risk of cardiopulmonary collapse. Especially since the investigated patient population may not live long enough to experience the favourable prosthesis-related outcomes associated with cemented hemiarthroplasty, such as reduced risk of periprosthetic fractures [3, 8, 24, 25]. However, this study did not find a statistically significant association between the use of cement and in-hospital mortality, which contradicts earlier literature that has linked cement use to short term mortality [5, 11]. Cardiopulmonary collapse and mortality can have other underlying systemic causes and are observed in frail patients undergoing any type of surgery. This might explain why we did not find an increased risk of in-hospital mortality after cemented hemiarthroplasty in this especially frail study population, while studies investigating the general population treated with cemented or uncemented hemiarthroplasty did. Another explanation might be that the statistical power of this study was too low to show that the estimated sixfold increase of in-hospital mortality after cementation in this frail patient group was statistically significant.

Many of the previously identified risk factors for cardiopulmonary collapse contribute to or point towards a poor cardiac function [4, 5, 8]. In this study, cardiopulmonary collapse was three times as likely in frail FNF patients if they had an abnormal LVEF. It is therefore advisable to preoperatively assess patients at risk for severe BCIS with an echocardiography if they are indicated for hemiarthroplasty. If patients demonstrate a decreased LVEF, perioperative management with the emphasis on euvolemia, avoidance of excessive pressurization and extra consideration of the indication for a (cemented) hemiarthroplasty [26, 27].

In this study, patients with a CVA or TIA in the medical history of patients showed a reduced risk for cardiopulmonary collapse. Standard care after enduring a CVA or a TIA is prophylactic anti-coagulants. Although anti-coagulants administration is halted before hip fracture surgery, it is imaginable that the effects endure till and even after fixation of arthroplasty. This anti-coagulant effect might have beneficial systemic effects that may influence the risk of cardiopulmonary collapse. This result however is in variance with the literature and should be interpreted with care. One recent study reported a significant increase of BCIS in patients treated with heparin compared to without, another study reported a decrease of BCIS in patients treated with warfarin. [4, 5].

Approximately 30% of the patients with femoral neck fracture treated with cemented hemiarthroplasty met the inclusion criteria of this study, being aged ≥ 80 years, ASA ≥ 3 and at least one cardiac or pulmonary comorbidity. Further efforts should be made to identify the frail patient population, for whom the risk of cardiopulmonary collapse associated with cemented hemiarthroplasty warrants extra consideration when determining the indication of a hemiarthroplasty.

### Limitations

Data quality may have been affected by the retrospective study design. The baseline blood pressure, used for the calculation of the grade of BCIS (Table 1), was the first measured blood pressure in the operation room. However, relying on a single value is susceptible to bias by e.g., pain induced hypertension. To limit information bias, the extraction of data from the medical records in both hospitals was performed by the same investigator. Due to the observational nature of the study, the selection of patients for hemiarthroplasty was not protocolized but at the discretion of the treating surgeon. However, fixation with or without cement was routine standard of care in both hospitals. Baseline differences between the cohorts were dealt with in the analysis by adjusting for potentially relevant confounders. Many of these factors can be attributed to logistic and organizational differences between the two centers. Still, we adjusted for population differences after data collection using univariate and multivariate analyses. A multicenter RCT on this topic, to overcome all of the mentioned limitations, will be challenging considering the inclusion criteria and the difficulties of obtaining informed consent in frail older trauma patients; 48% of all included patients had dementia (Table 2). Additional to that, ethical considerations concerning the in-hospital mortality risk of cementation apply. A prospective study that studies the predictive value of the LVEF for BCIS in patients treated with cemented hemiarthroplasty using preoperative echocardiography seems feasible and could contribute to the risk assessment and management of BCIS.

Since a formal sample size calculation was not possible due to absence of relevant data in literature, interpretation of the results of this study should be done with care.

## Conclusions and implications

This study indicates a threefold risk for perioperative cardiopulmonary collapse in frail FNF patients treated with cemented hemiarthroplasty compared to those treated with uncemented hemiarthroplasty. Also, an abnormal left ventricle ejection fraction is a risk factor for cardiopulmonary collapse in frail hip fracture patients treated with cemented and uncemented hemiarthroplasty. We therefore underline the importance of intra-operative euvolemia and avoidance of excessive pressurization, and careful consideration of an indicated (cemented) hemiarthroplasty in frail hip fracture patients. Further research is required to substantiate the frail population at risk of cardiopulmonary collapse after hemiarthroplasty.

## Data Availability

No datasets were generated or analysed during the current study.
